# Complexity reduction and opportunities in the design, integration and intensification of biocatalytic processes for metabolite synthesis

**DOI:** 10.3389/fbioe.2022.958606

**Published:** 2022-07-22

**Authors:** Roland Wohlgemuth, Jennifer Littlechild

**Affiliations:** ^1^ Institute of Molecular and Industrial Biotechnology, Lodz University of Technology, Lodz, Poland; ^2^ Swiss Coordination Committee for Biotechnology, Zurich, Switzerland; ^3^ Henry Wellcome Building for Biocatalysis, Biosciences, University of Exeter, Exeter, United Kingdom

**Keywords:** cell-free biocatalysis, enzyme development, enzyme function, reaction engineering, biocatalytic process design, metabolite synthesis, bioprocess navigation

## Abstract

The biosynthesis of metabolites from available starting materials is becoming an ever important area due to the increasing demands within the life science research area. Access to metabolites is making essential contributions to analytical, diagnostic, therapeutic and different industrial applications. These molecules can be synthesized by the enzymes of biological systems under sustainable process conditions. The facile synthetic access to the metabolite and metabolite-like molecular space is of fundamental importance. The increasing knowledge within molecular biology, enzyme discovery and production together with their biochemical and structural properties offers excellent opportunities for using modular cell-free biocatalytic systems. This reduces the complexity of synthesizing metabolites using biological whole-cell approaches or by classical chemical synthesis. A systems biocatalysis approach can provide a wealth of optimized enzymes for the biosynthesis of already identified and new metabolite molecules.

## Introduction

The quality of human life has continued to benefit from the rapid development of new medicines for the treatment of many diseases. The tremendous growth of knowledge in the discovery, isolation, structural characterization and man-made synthetic replication of small molecules formed naturally in living organisms represents a new domain of synthetic biology. The man-made synthesis of the metabolites has been of fundamental importance in this area. The first demonstration of this approach was using urea or acetic acid characterised as organic molecules from living organisms that could be synthesized from inorganic starting materials. Today this has developed into the total synthesis of metabolites having the most complex molecular structures (Nicolaou et al., 2000; Nicolaou and Rigol, 2020). The development of broadly applicable synthetic methodologies for the construction of the carbon backbone and for the selective introduction of a large diversity of functional groups, has created the necessary knowledge base for human creativity to find sequences of reactions for targeting products that can be prepared from available starting materials (Woodward, 1966; Eschenmoser and Wintner, 1977; Corey, 1991; Eschenmoser, 2015; Nicolaou and Rigol, 2020). As chirality is a key feature of the small molecules of living organisms, the analysis and synthesis of the absolute configuration of the metabolites involved in the metabolic pathways of interest are of central importance. The synthetic methodologies rely either on the introduction of new chiral centers into the starting materials by asymmetric synthesis or by the utilization of bio-based starting materials with the naturally occurring stereochemical configuration such as the chiral pool of compounds, including amino acids, carbohydrates, or terpenes. Apart from the selection of the most suitable and easily available starting materials and a classical primary criteria for evaluating a synthetic route, such as yield, number of reaction steps, safety, health, environment or cost aspects, there are additional factors that have an influence on what is considered as the ideal synthesis of a given target compound (Gaich and Baran, 2010; Wender 2014; Peters et al., 2021). Highly chemoselective reactions which do not require the initial introduction of protecting groups and their later removal, not only reduce complexity and the waste generated, but are also a clear goal to provide support towards the ideal synthetic process (Young and Baran, 2009). The total time needed to prepare a metabolite from the starting materials available can be the predominant determining criterion for determining the ideal synthetic route (Hayashi, 2021). This can either be man-made in the laboratory or using the metabolic pathway adopted by biological organisms or a combination of both.

The goals of synthetic chemistry towards the ideal synthesis in the laboratory can be greatly facilitated by the metabolomic information available from the highly evolved thousands of biocatalytic reactions that occur naturally in biological cells. This provides a complimentary and fruitful interface for making use of “natures enzymes” to catalyze protecting-group free reactions with reduced complexity and minimal waste. The development and application of biocatalysis is therefore key to the science of small molecule synthesis and is enabling the move towards sustainable synthetic routes that contribute to the UN Sustainable Development Goals and the European Green Deal.

The first man-made synthesis of a metabolite occurring in living organisms from inorganic starting material (Wöhler, 1828), namely the synthesis of urea outside the kidney from ammonium cyanate, represented a milestone achievement and is of fundamental interest. From that time until today great advances have enabled the development of a large variety of synthetic processes for valuable metabolites. From the beginning, the question of fossil or bio-based starting materials can be illustrated by the initial development of the coal tar based chemical synthesis of indigo (Baeyer, 1900), which took over industrial manufacturing leading to a large increase in synthetic indigo production from petrochemicals, whereas the natural plant-based indigo production decreased. While economic determinants such as overall production costs are still prevailing, the increasing importance of health and environmental aspects are changing the boundary conditions and have revitalized the interest in bioprocesses towards one of the oldest and most often used indigo dyes (Mendoza-Avila et al., 2020). In addition, the industrial large-scale manufacturing of metabolites today needs to deal with new boundary conditions and increased attention to the safety, sustainability of the involved reactions and chemicals, industry sector-specific strategies and customer preferences for biobased starting materials. These conditions as well as numerous other factors, solubilities, thermodynamics, kinetics, downstream processing, product recovery and purification, have an influence on the final process design.

Biocatalysis and the exploration of its frontiers have inspired numerous biocatalytic process designs with high molecular economy (Wohlgemuth 2021; Alcántara et al., 2022). These have been realized in various ways, ranging from single step reactions to their combination with other biocatalytic or chemocatalytic reactions. This can be achieved by a variety of methods ranging from the use of purified enzymes or cell-free extracts, to the organization of the required enzymes in multi-enzyme assemblies both *in vitro* or *in vivo* using engineered whole-cells.

## Synthetic access to the metabolite and metabolite-like molecular space

**FIGURE 1:**
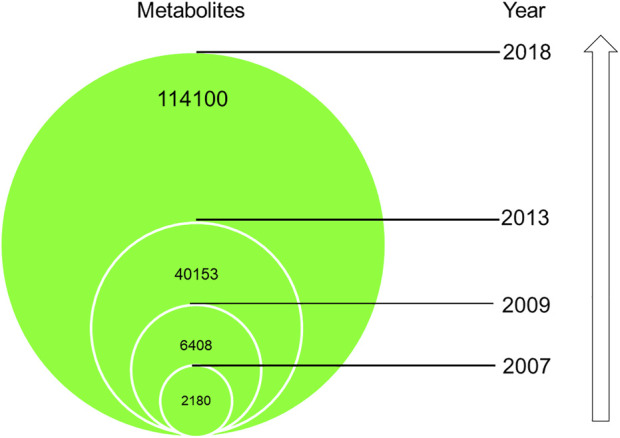
A diagrammatic representation of the growth of described metabolites from 2007 in the Human Metabolome Database (HMDB) as reported by Wishart et al. (2018).

The interest and knowledge in the total metabolite molecular space of a biological organism, the metabolome, and the related metabolite-like small molecules interacting with enzymes has increased tremendously over the past 15 years. The metabolomes of biological organisms are of key importance together with its genomes, transcriptomes and proteomes. This is very clearly illustrated by the growth of the world’s largest organism-specific metabolome database, the human metabolome database HMDB (Figure 1). The first release, HMDB 1.0 described 2,180 metabolites and its fourth release increased to 114,100 metabolites (Wishart et al., 2018).

Although the synthesis of metabolites goes back to the origins of organic chemistry and to the historic milestones in biochemistry, the actual gap between the number of known metabolites and the ones for which the synthesis has been reported is widening (Wohlgemuth, 2018). The question of why there is this widening gap and why the synthetic access to natural metabolites and metabolite-like molecules is important and relevant beyond the fundamental aspects of the science of synthesis, is connected with different areas of the life sciences, including biomedicine and industry. Having sufficient amounts of pure metabolites or metabolite-like compounds is essential in the analysis and identification of metabolites for the areas of metabolomics, enzyme assays, and discovery of novel biological functions. Enantiomerically pure metabolites are key to the precise kinetic characterization of enzymes and understanding their substrate enantioselectivity.

Chiral metabolites are more challenging to synthesize by traditional synthetic chemical methods, which often involve many steps including protection and deprotection of functional groups due to non-selective synthetic methods. Nature synthesizes chiral metabolites naturally in specific stereochemical configurations since enzymes themselves are chiral and are made up of L-aminoacids. This property means that enzymes will usually only use one enantiomer of a specific substrate. However some enzymes do show promiscuity especially when non-natural substrates are used in biocatalytic synthetic reactions. It has been shown that a change in L to D configuration in specific amino acids within a protein can result in its misfolding. When the protein structure cannot fold correctly this can lead to the production of amyloid deposits that cannot be degraded by natural proteases, so they accumulate in specific tissues such as the eyes and the brain. Specific racemase enzymes have been found in human tissue which can be responsible for this change (Ohide et al., 2011). The analysis of the chiral identity of metabolites is a powerful biomarker for monitoring of human disease and is an area for development within clinical medicine. The importance of being able to have standard metabolite molecules to identify these changes is of increasing importance. d-amino acids are naturally found in bacterial cell walls and are therefore associated with our microbiome (Bastings et al., 2019). The importance of understanding of an individual’s microbiome and its symbiotic importance to human health is a topical research area. It is known that our microbiome is involved in immunity and the function of the gut barrier (Ruth and Field, 2013).

The synthesis of metabolites is essential in the development of new chemical entities intended for interacting with biological organisms, such as drugs for human and veterinary use or agrochemicals for plant protection. As the multitude of metabolites in a biological cell is achieved through a variety of natural biocatalytic reactions, the reduction of the complexity in the synthesis of a desired single metabolite can be carried out using a systems biocatalysis approach. This can use only the required biocatalytic reactions in cell free biocatalysis (Schmid-Dannert and López-Gallego, 2019; Bergquist et al., 2020; Sutiono et al., 2021), use cell-free biosynthesis (Morgado et al., 2016), use artificial metabolism (Fessner, 2015) or synthetic metabolism (Erb, 2019), all providing attractive strategies for the design of new biocatalytic processes for metabolite synthesis. The substrate scope of metabolic enzymes provides also an opportunity for the synthesis of metabolite-like molecules, which are of interest for potential new pharmaceuticals, since a major fraction of approved drugs have been shown to have a similarity to metabolites (Swainston et al., 2015). Although the importance of metabolites and metabolic pathways in connection with disease has long been known, it is only through recent scientific advances and discoveries that metabolites have attracted much attention, for example in metabolites as signatures of altered cancer cell metabolism associated with specific types of cancer (Ward and Thompson, 2012). Certain cancer-specific metabolites which are no longer seen as an indirect response to cancer cell proliferation but have themselves been shown to be oncogenic, have been designated as onco-metabolites (Leca et al., 2021).

## Search and selection of optimal route, starting materials and biocatalysts

**FIGURE 2:**
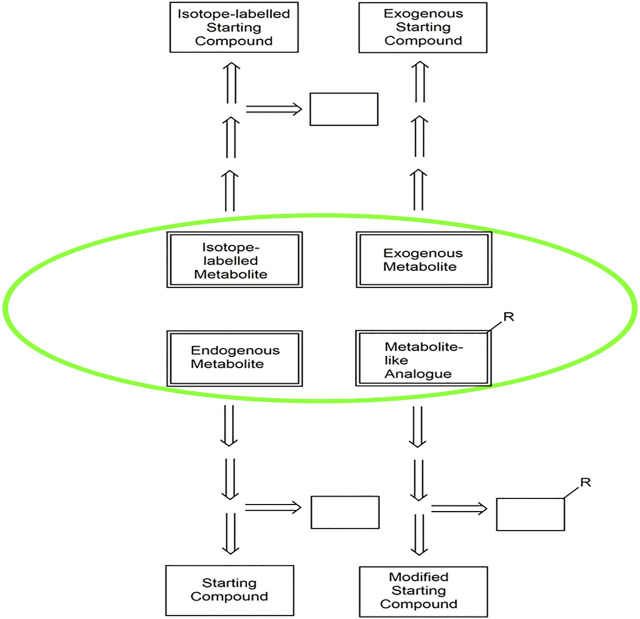
An overview of the selection of synthetic routes for endogenous metabolites, exogenous. metabolites, isotope-labelled metabolites and metabolite-like analogues, highlighted in. green. The arrows indicate the retrosynthetic pathways from the different classes of. metabolites to the starting compounds. The open boxes represent additional auxiliary. molecules in the different pathways.

A key success factor for facile synthetic access to metabolites is the identification of the optimal route to the target molecules (Figure 2), whereby database, experience and retrosynthetic analysis tools in both the biochemical and chemical domains can provide valuable guidance. Suitable starting materials resulting from the disconnection of bonds of the target molecules in the retrosynthetic analysis can be evaluated according to criteria such as commercial availability, cost, fossil or biobased origin.

The starting materials for metabolite synthesis are an important consideration, also with respect to the reduction of complexity, because starting from highly reduced fossil-based starting materials the synthetic route to the metabolite can involve many more reaction steps and be more challenging than a straightforward defunctionalization reaction from a biobased starting material containing already many oxygen- and nitrogen-containing functional groups (Wohlgemuth, 2022).

The selection of suitable starting materials depends on various factors, such as availability, quality, stability, customer requirements, safety, health and environment aspects, economic conditions, or the supply chain. The stability of the supply chain in the long term, amid needs and sustainability considerations may also affect the choice of fossil-based versus biobased starting materials. A number of parallel developments are supporting research and development on the transition from fossil-based to biobased starting materials. The number and molecular diversity of biobased starting materials is continuously increasing, with many classical compounds already been traditionally manufactured from bioresources. Top platform chemicals have been identified, starting in 2004 by the United States Department of Energy (Werpy and Petersen, 2004) and updated in 2010 (Bozell and Petersen, 2010). Many more platform chemicals derived from bioresources are becoming available as biobased starting materials and new compounds derived by novel routes from bioresources are being developed (Jang et al., 2012; Choi et al., 2015). In order to develop syntheses using biobased resources which can replace petrochemicals, knowledge of the production of the starting material needs to be considered. A globally standardized and simple descriptor for the source of each compound is not only facilitating the clear choice between fossil-based and biobased starting materials, but is also beneficial for quality purposes.

Examples of biobased starting materials which are available at industrial large scale and can be considered as a substitute for the corresponding fossil-based starting materials can include a mix of older and newer compounds. These could be ethanol and related alcohols, 1,3-propanediol, glycerol, sugar alcohols like sorbitol and xylitol, organic acids like acetic acid, 3-hydroxypropanoic acid, lactic acid enantiomers, levulinic acid and succinic acid, furans like furfural, 5-hydroxymethylfurfural, and furan-2,5-dicarboxylic acid, or hydrocarbons like farnesene, isoprene and limonene. Matrices and biological materials which can be used as biobased starting materials are mainly derived from natural photosynthesis, which provides inexpensive and renewable polysaccharides and biomass.

The move away from fossil based starting materials is being driven by the push towards a sustainable circular bioeconomy. With this in mind the optimal route and starting materials should be determined by the search and availability of the optimal biocatalysts. Enzymes have traditionally been classified into six main classes which is based on the type of reaction that they catalyze. This first classification was introduction by Dixon and Webb and was establishment as a fundamental enzyme classification scheme by the enzyme commission more than 60 years ago (McDonald and Tipton, 2022). However a major change of the system was introduced by the enzyme commission in 2018 with the establishment of a new seventh class named as translocases describing enzymes catalyzing transport across cell membranes (McDonald and Tipton, 2022). The concept of enzyme engineering for optimization of a particular process is now generally accepted. Hence a key factor is the initial selection of the most suitable biocatalysts, that can carry out the desired reactions in a highly selective, sustainable and energy efficient manner.

Gene sequencing techniques have rapidly developed in the last few years allowing the genomes of many animal, plant and microbial species to be available for identification of a host of new enzymes which can be characterised and produced for different metabolite synthesis. In addition the concept of metagenomics has allowed DNA to be isolated and sequenced from different unique environments. It is estimated that we can only cultivate 5% of the existing microorganisms found in nature so metagenomics allows identification of a wealth of new enzymes which would have escaped our current knowledge. New genes can be identified using a bioinformatic approach to search for a known class of enzyme using the amino acid sequence and known protein structural motifs. These motifs identified in known proteins are often specific for the binding of ATP and cofactors necessary for enzyme activity which can help with annotation. Protein structural space is restricted to a limited number of overall folds (arrangement of α-helices and β-sheets) that can be used in different proteins with a variety of different catalytic activities which can make the specific identification of many proteins difficult. However the increasing number of protein structures deposited in the Protein Data Bank, which currently exceeds 180,000, together with new improved modelling techniques based on artificial intelligence such as Alphafold2 (Jumper et al., 2021), have increased the amount of protein structural information available.

The use of expression libraries generated from available DNA sequences is another approach to identify new enzymes, which relies directly on enzyme activity. The DNA is cleaved into “gene size” fragments and inserted into expression plasmids which can be induced to over-express a library of many different gene products in a suitable host such as *Escherichia coli*. This library can be screened to identify single bacterial colonies that are able to grow on the selected substrate of interest by either direct visualisation of clearing zones on agar plates containing the substrate of interest or by colorimetric identification specific for a particular product. This technique allows the identification of enzyme activities against non-natural substrates and can identify different enzyme classes that are active against a particular substrate for example racemic γ-lactam (Taylor et al., 1993).

The metabolic pathways and genes are easier to identify from bacterial species since they are often encoded in tandem within the genome allowing one messenger RNA (polycistronic mRNA) to be expressed when the whole enzyme synthetic pathway is required by the microorganism. Also many bacteria have a secondary metabolism that it only turned on in response to different growth conditions hence it is important to consider genomics and proteomics. In addition to genomic DNA many bacteria carry genes on large plasmids which enable them to grow under specific conditions such as in the presence of camphor (McGhie and Littlechild, 2017).

In eukaryotes the genes within in a metabolic pathway are often more difficult to identify since they can be distributed over the genome and each gene contains several introns (uncoded regions) that have to be removed from the mature mRNA before it leaves the cell nucleus. Molecular biology techniques are used to copy the mature messenger mRNA to DNA using a viral reverse transcriptase enzyme to produce cDNA that can be cloned directly using suitable host systems such as *E. coli*. The cost and availability of synthetic gene synthesis now allows the rapid cloning of codon optimised DNA sequences which can be tailored to a chosen expression host. Advances in genome annotation and improved bioinformatic techniques will continue to allow the identification of new novel biocatalysts for metabolite synthesis.

As there can be a mismatch between the reaction requirements and the enzyme properties and performance, regarding activity, stability or enzyme inhibition, this can cause a problem. However enzyme engineering approaches provide a powerful tool for the optimization of enzyme performance. The enzymes can be evolved and optimised for the reaction conditions by directed evolution (Reetz, 2016; Arnold, 2018; Chen and Arnold, 2020) or rational protein engineering methods that are directed by the structural knowledge of the enzyme of interest and an understanding of its mechanism. Enzymes can also be identified from organisms that grow naturally at high temperatures which have evolved to be more robust and stable to extreme conditions and as such are better for industrial processes (Littlechild, 2015a,b; Littlechild, 2017).

While this enzyme optimization applies to both whole-cell and cell-free approaches, the effective contribution of metabolic engineering differs in whole-cell systems, cell-free lysates and cell-free systems using purified enzymes. While the blocking or repression of an enzyme which catalyzes the further conversion of the metabolite in whole-cell systems is a well-established methodology, the large diversity of other enzymes in whole cells increase the likelihood of diverting the desired metabolite to by-products, which may be difficult to separate due to similar properties as the desired metabolite. Therefore, the contribution of metabolic engineering for optimizing the flux to the desired metabolite and reducing unwanted by-product formation is very important.

Enzyme optimisation for *in vitro* synthesis can be directly carried out with each enzyme optimised for stability and/or substrate specificity based on its structure and mechanistic properties. Other desired properties can be introduced using directed evolution as mentioned above. If whole cells are used for an *in vivo* synthesis using a synthetic cascade of enzymes this can be more challenging, however the designed cascade can be introduced into the host cells using a suitable plasmid and promotors for each individual enzyme. This can be approached using BioBricks cloning methodology (http://hdl.handle.net/10871/27323) where different genes are assembled with their own promotors within an expression vector. This is easier to carry out if a natural pathway is to be used, for example the synthesis of the natural extremolyte molecule 2,3-diphospho-d-glycerate that can protect cellular components to high temperatures (De Rose et al., 2021). However if a new synthetic pathway is designed problems of flux need to be addressed. Genetic manipulation of the host organism is often required to overcome these problems. It has been possible to alter the steady state flux in a cell by knockout of specific genes, replacing promotors and introduction of heterologous genes in a systems metabolic engineering approach (Brockman and Prather, 2015; Ko et al., 2020). Other approaches involve so called “precision engineering” where the design and engineering of systems use a cellular response to specific signals to optimise production strategies (McNerney et al., 2015). Computational modelling can address different problems in optimising metabolite production, for example control of enzyme expression, toxic pathway intermediates, and product inhibition of upstream enzymes.

Fast and meaningful experimental analytical assays are a key success factor for all types of enzyme optimizations. Analytical advances in droplet-based microfluidics and ultrahigh-throughput screening methods have led to practical benefits for rapid enzyme improvements (Colin et al., 2015; Bunzel et al., 2018; Gielen et al., 2018). Tremendous methodological advances in MS and NMR have enabled the combining of high information content with simplified, fast and sensitive analysis. The development of desorption electrospray ionization (DESI) mass spectrometry (Takáts et al., 2004) has provided a powerful analytical technology for rapid and direct analysis of enzymatic reactions without having to use labels (Morato et al., 2020; Morato et al., 2020; Kulathunga et al., 2022). Another powerful label-free analytical technology has been demonstrated by using micro-fluidics to sort nanoliter droplets whereby the droplets are split with direct detection by ESI-MS on one side, and on the second side with sorting according to the result of the ESI-MS analysis (Holland-Moritz et al., 2020).

Advances in enzyme immobilisation techniques (Sheldon et al., 2021) have provided a cost-effective and sustainable way of using enzymes as recyclable heterogeneous catalysts, which are easier to be removed from the bioreactor vessels by either centrifugation or filtration.

## Cell-free versus whole-cell biocatalytic systems

**FIGURE 3:**
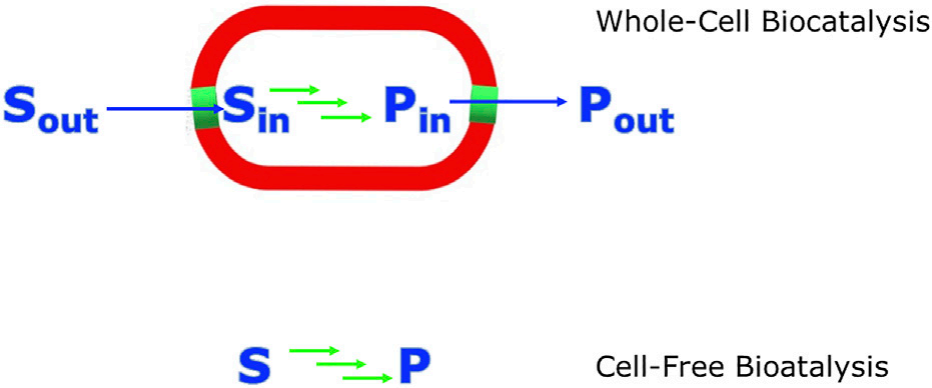
A schematic comparison of biocatalytic synthesis in whole-cell versus cell-free systems. The green arrows indicate the biocatalytic reaction steps involved. The blue arrows. indicate the transport of substrates and products across the cell membrane.

While the use of whole cells for producing metabolites has a long history, the use of cell free biocatalytic systems has attracted increasing interest over the last 20 years (Hodgman and Jewett, 2012; Swartz, 2018). To carry out biocatalytic reactions outside a living cell (cell-free, *in vitro*) the respective enzymes need to satisfy certain requirements, such as stability for the synthetic process. The thermodynamics and kinetics of natural pathways can facilitate the design of similar new bioprocess learning from naturally evolved flux of the enzymes which allow the pathway to flow in the desired direction. For newly designed synthetic and non-natural pathways where the energetics and flux is not favourable suitable adaptations need to be designed considering both thermodynamic and kinetic aspects. Metabolic engineering of whole-cells as well as cell-free systems can allow the choice of enzyme activities and their sequential arrangement to allow a favourable flux to drive the system to completion. Some interventions are much simpler for cell-free systems, for example precise coordination and temporal control of the reactions in a sequence by modifying the quantities of the enzyme catalysts used in relation to their different turnover rates. However this is more challenging in whole cell systems since the enzymes converting precursors into the desired metabolite need to be optimized for their increased and coordinated expression. At the same time enzymes diverting precursors towards side products need to be repressed (Zalatan et al., 2015). A variety of molecular and engineering tools can be utilized for both whole-cell and cell-free systems to provide more favourable energetics. As whole-cell and cell-free systems (Figure 3) have their advantages and disadvantages the design of a hybrid bioprocess could divide the parts of a bioprocess which are best supported by a whole-cell and those by a cell-free system. The choice of the system depends on various boundary conditions which may also change over time such as economic aspects (Claassens et al., 2019).

If the process of biosynthesis is carried out *in vivo* by a natural pathway or the construction of synthetic enzyme cascades/pathways in whole cells, the cellular environment provides some protein stability and also the vital co-factors that are required for the activity of many of the natural enzymes. On the other hand this *in vivo* approach also requires that there is effective transport of the required substrates into the whole cells. In addition the transport of the synthesized metabolite products out of the whole cells needs to be optimized to avoid mass transport limitations, or the whole cells need to be disintegrated before the metabolites can be isolated and purified. The efficient and selective transport of products through the cell membranes out of the cells can be an advantage for the use of whole-cell systems. However the energetics of having in addition to the desired bioprocess, the need to sustain various other processes in whole-cells and the kinetics of the reactions and transport issues may limit the efficiency of the whole-cell bioprocesses. Another challenge for the use of whole cells in metabolite synthesis is the need to genetically delete enzymes which catalyze undesired side reactions of substrates, intermediates and products, thereby decreasing the efficiency of the bioprocess.

From the perspective of complexity reduction, carrying out a biosynthetic process *in vitro*, or *ex vivo*, as described over 25 years ago (Roessner and Scott, 1996), can overcome limitations of whole-cell approaches by the opportunity to focus only on the required reactions for optimizing productivity, diversity or new-to-nature metabolites. The use of cell-free systems, whether in the form of a crude cell lysate or composed of a number of isolated and purified enzymes, has some fundamental advantages compared to whole cell systems. This approach gives the opportunity to focus only on the required reactions and their optimization towards high productivity by avoiding product losses by unwanted side reactions, bypassing complex interactions of substrates, intermediates or products resulting in toxicity to whole cells, and simplifying product recovery and purification as the cell membrane/cell wall and complex cellular components are removed. The first demonstration of cell free biocatalytic processes was carried out in the 19th century by Eduard Buchner, who received the 1907 Nobel Prize in Chemistry “for his biochemical researches and his discovery of cell-free fermentation” (Buchner, 1897; Buchner 1907). Great advances have enabled the assembly of recombinant enzymes in a modular fashion, such as in the biocatalytic synthesis of metabolites of the natural aerobic and anaerobic vitamin B_12_ pathways using cell-free systems (Scott, 2003). Reproducible and stable biosynthesis of metabolic intermediates at submicromolar scale was the reason for moving from whole cell preparations of *Propionibacterium shermanii* to the cell-free synthesis of corrin metabolites such as cobyrinic acid from uroporphyrinogen III and aminolevulinic acid (Scott et al., 1973). The cell-free biocatalytic synthesis of the corrin metabolite hydrogenobyrinic acid from aminolevulinic acid, which uses 12 overexpressed enzymes for catalyzing 17 reaction steps in one pot, has been achieved with an overall yield of 20% (Roessner et al., 1994; 1995). A cell-free system containing nine enzymes, including auxiliary enzymes and cofactor regenerating enzymes, enabled the one-pot synthesis of nepetalactol or nepetalactone starting from geraniol at a much higher product concentration of about 1 g L^−1^ than the highest titer described for microbial systems (Bat-Erdene et al., 2021). The use of cell-free systems is highly attractive due to its modularity and continues to attract much interest in cell-free metabolic engineering (Dudley et al., 2015; Swartz, 2018) and synthetic biochemistry (Bowie et al., 2020).

## Design of biocatalytic processes

Before starting the design of a biocatalytic process in the context of metabolite synthesis it is important to define the design goals. The most common design goal is to find the optimal synthetic route towards a desired metabolite in a target-oriented synthesis. There may however be other situations where the design goal is to find suitable targets which can be synthesized from a common starting material in a diversity-oriented synthesis, or to reduce complexity towards obtaining a compound most suited for a particular application in a function-oriented synthesis. While in target-oriented synthesis a major reason for synthesizing natural products has been to provide simplified access to the desired target product in a defined quality for its function, whereas the strategy of function-oriented synthesis is to achieve a desired function of the target product by design and with synthetic economy (Wender et al., 2008; Wender et al., 2015). Bioactive small molecules from natural resources are of great value for drug development and medicine, but simplification of their structural complexity by removing unnecessary or even disturbing substructures can overcome bottlenecks and limitations of their drug properties, pharmacokinetics, and synthesis (Wang et al., 2019).

**FIGURE 4:**
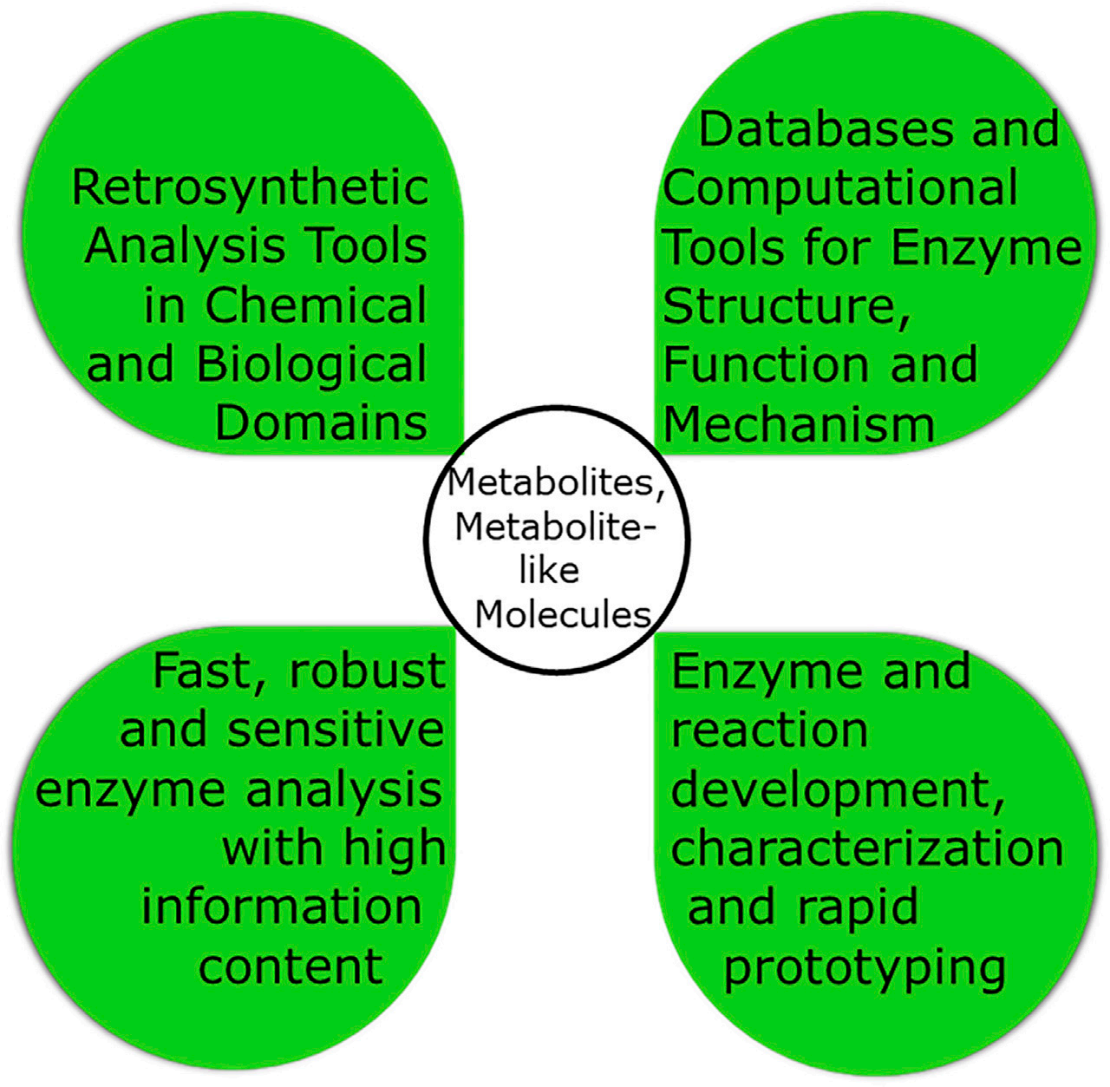
The four key aspects for the design of a biocatalytic process in target-oriented. synthesis of metabolites and metabolite-like molecules.

This paper will concentrate on target-oriented synthesis for which Figure 4 highlights a number of key design tools. In this synthetic approach, retrosynthetic analysis in the chemical and biological reaction space provides the background for navigating through the various routes which can be thought of from starting material to the product. Considering molecular economy aspects together with the lowest number of reaction steps is important for integrating multiple steps. Finally process intensification not only for the reaction but also for the product recovery and purification is essential for practical implementation. While biocatalytic process design is a complex task the increased experience with the innovation steps for successfully implemented bioprocesses provides an excellent practical knowledge base for innovation in systematic process design methods and for integrating various methodologies, such as protein and process engineering (Woodley, 2017).

Retrosynthetic analysis from both the biological as well a chemical perspective provides a design framework with which the most promising solutions can be used for rapid prototyping and obtaining a proof of principle (Wohlgemuth, 2017). The analysis of chemical and biological routes for the synthesis of metabolites often shows large differences in the number of reaction steps required to construct highly and differentially functionalized small molecules. Designing highly selective and straightforward biocatalytic one-step synthesis methods instead of lengthy and challenging chemical routes has been established as the preferred method for preparative access to a large number of valuable metabolites (Richter et al., 2009; Schell et al., 2009; Matsumi et al., 2014; Schoenenberger et al., 2017a; Hardt et al., 2017; Schoenenberger et al., 2018; Krevet et al., 2020; Schoenenberger et al., 2020; Sun et al., 2021). As the finding and selection of routes is a key task in target-oriented synthesis the development of appropriate methodologies has attracted much interest in both chemistry and biology. New tools for retrosynthetic analysis have been developed both in the domain of biochemical (Delépine et al., 2018; Lin et al., 2019; Hafner et al., 2021; MohammadiPeyhani et al., 2022) as well as chemical reactions (Mikulak-Klucznik et al., 2020).

Suitable methods with the necessary high information content, such as MS and NMR, for the analysis of the biocatalytic transformations and products are therefore a key success factor, both for the development and in-process control in the production phase (Jani et al., 2008; Gauss et al., 2014; Matsubara et al., 2014; Schoenenberger et al., 2017b).

The complexity of the downstream processing, product recovery and purification steps can be greatly reduced if simple purification methods such as crystallization, precipitation, extraction, phase separation, or distillation, which have been successfully designed for one metabolite, can be transferred to other metabolites of the same class (Wohlgemuth, 2011).

## Integration of biocatalytic reactions

**FIGURE 5:**
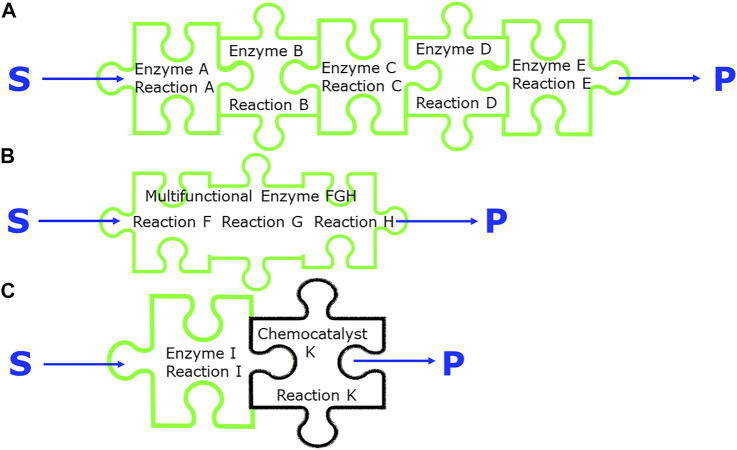
Different integration strategies of biocatalytic processes, **(A)** use of five different enzymes. in a sequential reaction cascade or simultaneously in a one-pot reaction, **(B)** use of one multifunctional enzyme catalyzing three related reactions, **(C)** use of a biocatalytic and a chemocatalytic reaction in a sequential reaction or in a one-pot reaction.

Biocatalytic reactions can be integrated along the paths of multiple biocatalytic reactions steps (Figure 5), or along biocatalytic and classical chemical reaction steps, or along the reaction, downstream processing and product recovery steps. Considering molecular economy aspects together with the lowest number of reaction steps is important for integrating multiple steps. The relevant aspects of molecular economy for step integration include the optimization of the step economy by reducing the number of reaction steps, by more direct transformations or by eliminating protection-deprotection schemes, by avoiding intermediate isolation and downstream processing steps through coupling several reaction steps in one pot. The examples of the manufacturing processes for mevalonate 5-phosphate (Matsumi et al., 2014), shikimate-3-phosphate (Schoenenberger et al., 2018), tagatose-1,6-diphosphate (Schoenenberger et al., 2020), 2-keto-3-deoxy-d-gluconate (Matsubara et al., 2014) and l-argininosuccinate (Schoenenberger et al., 2017a) demonstrate the importance of complexity reduction. This can be achieved by reducing the number of steps, avoiding protecting groups, selective biocatalytic phosphorylation reactions, selective defunctionalization and simple addition reactions reducing complex chemical multi-step routes.

Multiple biocatalytic reaction steps can be achieved by having a multifunctional biocatalyst catalyzing more than one reaction step, a principle used by nature, for example in case of the polyketide synthases and the non-ribosomal peptide synthetases (Adrover-Castellano et al., 2021). This is also of much interest for biocatalytic synthesis as demonstrated by the recent discovery of a multifunctional biocatalyst EneIRED, which can catalyse conjugate reduction, as well as imine reduction and reductive amination of a broad range of simple prochiral substrates to diastereomeric amines in one-pot (Thorpe et al., 2022). An already classical way of integration is the assembly of multiple biocatalysts acting in a reaction sequence, with each biocatalyst catalyzing one specific reaction step (Roessner and Scott, 1996; Cutlan et al., 2020).

There are many examples of enzyme cascades being effectively used in “one pot” reactions such as the production of plant benzylisoquinoline alkaloids using purified enzymes (Lichman et al., 2015; Zhao et al., 2018). The construction of process models is commonly carried out to help understand the enzyme reactions and their optimisation for scale-up (Finnigan et al., 2019). The biocatalytic conversion of 4-methyl toluate to 4-tolyl alcohol, which involved at least seven enzymes in three reaction steps and included cofactor recycling, has been optimized by the use and validation of process models based on characterized enzyme parts (Finnigan et al., 2019). An approach combining experimental enzyme kinetics with pathway modelling has been successfully applied to a reaction sequence for the preparation of 2-ketoglutarate from d-xylose, using five enzymes of the Weimberg pathway in a cell-free system (Shen et al., 2020). Linear biocatalytic reaction cascades have attracted increasing interest over the last years and offer excellent synthetic opportunities with the reduced complexity of designing biocatalytic reaction sequences from well-characterized single biocatalytic reactions operating in a simultaneous or sequential mode (Schrittwieser et al., 2018). In cases where some biocatalysts of a reaction sequence show only sufficient activities in whole cells, for example membrane-bound enzymes for which no active variant is available in soluble form, the combination of biocatalysts in purified form as well as in whole cells represents another integration opportunity. The plant metabolite *cis*-(+)-12-oxophytodienoic acid has been synthesized in good yield, high diastereoselectivity and low side product formation from linolenic acid by a cascade of three biocatalytic reactions involving lipoxygenase 13-LOX as an isolated enzyme and recombinant *E. coli* whole cells expressing the *Arabidopsis thaliana* genes encoding allene oxide synthase AOS and allene oxide cyclase AOC2 (Löwe et al., 2020). Integration of several biocatalytic reaction steps can be achieved by searching for a novel biocatalyst which is able to catalyze a more direct reaction route to the target product. It is important to know all of the enzymes in the natural biosynthetic route to a metabolite and any missing enzymes need to be identified (Caputi et al., 2018).

While natural pathway enzymes provide within their substrate scope facile synthetic access to metabolites or metabolite-like molecules, more diversified and non-natural metabolite-like compounds can become accessible by broadening of the substrate by enzyme engineering, by introducing new catalytic activities with non-natural substrates and by developing new chemical transformations by combining chemistry and protein engineering (Miller et al., 2022). The transformations which can effectively be performed by either a classical chemical reaction, a chemocatalytic or a biocatalytic reaction can enable the combination of the two worlds of classical organic synthesis and biocatalysis (Wohlgemuth, 2007; Roberts et al., 2010; Rudroff et al., 2018; Bering et al., 2022).

In cases where no enzymes are known to catalyze a particular reaction, the integration of chemocatalytic and enzymatic transformations in a one-pot process offers a synthetic opportunity, if mutually compatible reaction conditions can be found and any interfering cross-reactions and incompatibilities can be overcome. Regioselective C−H bond functionalization methods have been developed with halogenase enzymes and palladium-catalysed cyanation together with the incorporation of nitrile hydratase or nitrilase enzymes. This type of two- or three-component chemobiocatalytic system can be carried out in “one pot” at gram scale (Craven et al., 2021).

## Intensification of biocatalytic processes

**FIGURE 6:**
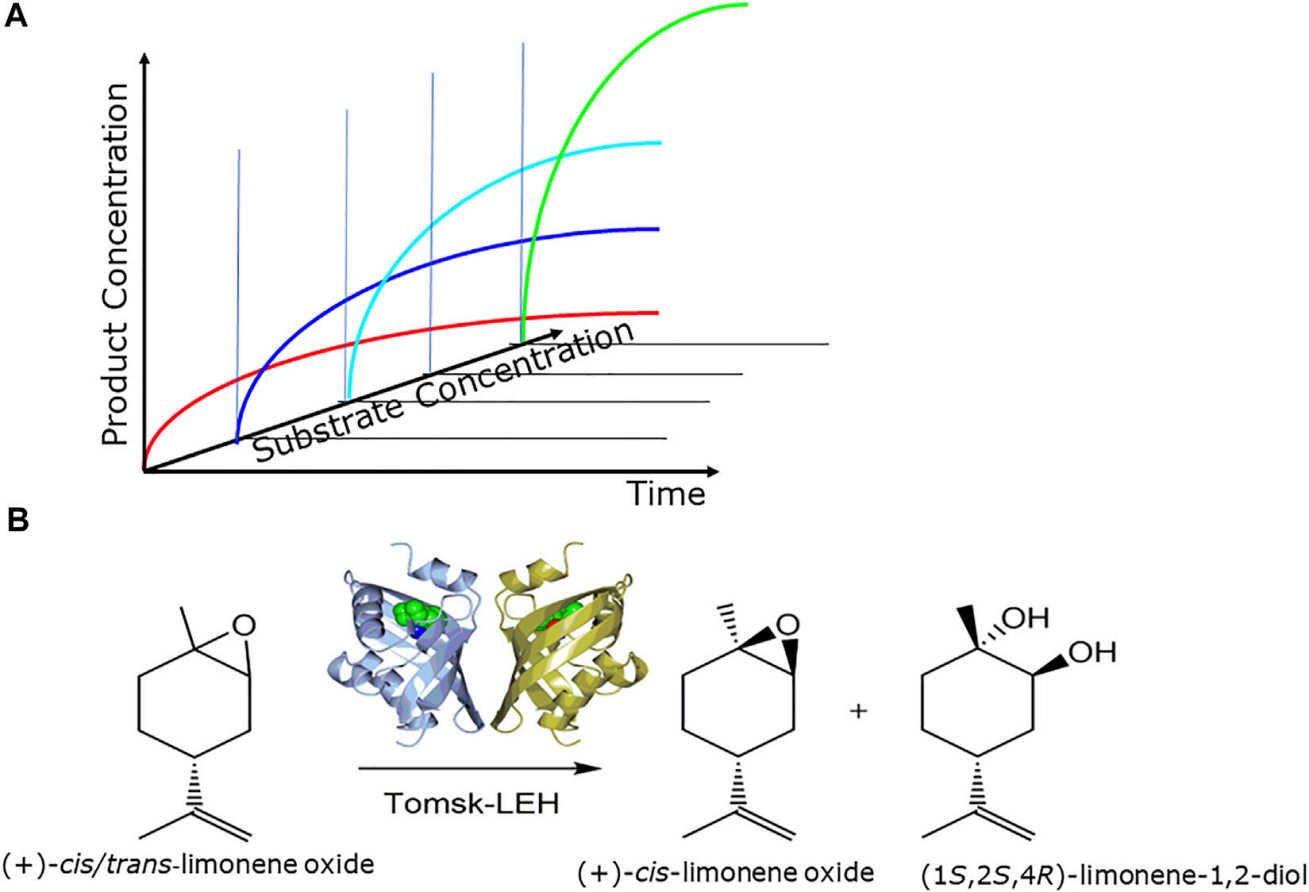
**(A)** Optimizing process metrics by increasing substrate concentration while maintaining complete conversion, **(B)** intensification of epoxide-hydrolase catalyzed resolutions of (+)-and (−)-cis/trans-limonene oxides, as illustrated for the Tomsk limonene epoxide hydrolase (LEH) for the production of (+)-cis-limonene oxide and the (1S,2S,4R)- limonene-1,2-diol (Ferrandi et al., 2015b).

The increase of the overall productivity of the prototype of the integrated biocatalytic procedure is essential for reaching a viable manufacturing process. The intensification of biocatalytic processes needs to take into account upstream, biocatalytic reaction and downstream phases (Boodhoo et al., 2022). The use of simple process metrics such as reaction yield, space-time yield, product concentration (Figure 6A) is thereby not only valuable for characterizing and comparing process performance in the course of intensifying, scaling and implementing biocatalytic processes, but also for setting targets for protein engineering and process engineering (Woodley 2017).

An important starting point for process development are the available process windows for favorable thermodynamics and stabilities of the components of the biocatalytic reactions, such as enzymes, substrates, intermediates and products, under the reaction conditions. While a variety of powerful methodologies are available for engineering the properties and stabilities of enzymes to match the optimum reaction conditions, the properties and stabilities of products can determine the reaction conditions, or otherwise product modifications need to be considered. In a next intensification phase biocatalytic reaction engineering aiming at complete substrate to product conversion and minimizing the formation of side products is an essential intermediate phase. In the final intensification phase the increase of the substrate concentration and the decrease of the enzyme usage is optimized for achieving the highest space-time yield, while maintaining the complete conversion and highest product purity from the previous intensification phase.

Overall biocatalytic process intensification builds on optimizing each biocatalytic reaction involved, since one single bottleneck in a biocatalytic reaction affects the entire process. This is illustrated with the biocatalytic process intensification for the synthesis of important chiral lactaldehyde and limonene oxide metabolites. The application of novel thermostable epoxide hydrolases (Ferrandi et al., 2015a). which are also stable in organic solvents, facilitates the use of poorly water-soluble substrates at high substrate concentrations. Complementary limonene-1,2-epoxide hydrolases have been applied in the process intensification up to 2 M substrate concentrations for the synthesis of all limonene oxide enantiomers as well as (1*S*,2*S*,4*R*)-limonene-1,2-diol and (1*R*,2*R*,4*S*)-limonene-1,2-diol (Ferrandi et al., 2015b) as illustrated in Figure 6B. The key ketoreductase-catalyzed asymmetric reduction steps in the synthesis of enantiomerically pure (*R*)- and (*S*)-lactaldehydes have been intensified in two development phases, whereby the substrate concentration could be increased from 6 to 250 g L^−1^, the cofactor concentration could be decreased from 0.8 to 0.05 g L^−1^, while maintaining complete conversion and excellent chemical and enantiomeric purity (Vogel et al., 2016).

Downstream processing methods with full product recovery in high quality are as important as the intensification of the reaction with complete conversion and are a key success factor for achieving viable processes with high overall resource efficiency. A variety of methodologies and tools are available for the intensification of metabolite downstream processing, recovery and purification (Wohlgemuth, 2009). Simple extraction optimization is illustrated by the examples of the complete extraction of enantiomerically pure 1,1-dimethoxy-2-propanols, which has been achieved by saturating the aqueous reaction solution with sodium chloride (Vogel et al., 2016). Working within process windows for product stability is essential in case of labile products for maintaining the product quality during downstream operations (Gauss et al., 2016). Simple product recovery and purification methodologies, such as phase separation, crystallization, or extraction, are attractive for intensification. However chromatographic separation may be needed, where simulated moving bed chromatography can be successfully used in the intensification of efficient separations of equimolar mixtures, such as enantiomerically pure regioisomeric lactones (Kaiser et al., 2009).

Process intensification over a convergent multistep reaction cascade in one pot has been demonstrated by the biocatalytic synthesis of MK-1454 (McIntosh et al., 2022). Process intensification phases involved classical optimization of reaction conditions, directed evolution of cyclic guanosine-adenosine synthase, optimizing cofactors and cosolvents, increasing substrate concentration, enzyme engineering of cyclic guanosine-adenosine synthase under optimized reaction conditions (Benkovics et al., 2022), which has intensified this biocatalytic process and improved the initial yield of less than 1%–91% (Benkovics et al., 2022).

## Discussion

The large “natural” reserve of known biocatalysts and the tremendous advances in tools and methodologies for designing, integrating and intensifying biocatalytic reactions are providing benefits and opportunities for future biocatalytic processes for the synthesis of metabolites and metabolite-like molecules. The high selectivity of natural biocatalysts removes the need for protection and deprotection reactions and reduces the complexity of functional group transformations thereby significantly simplifying the synthesis of more complex products. The metabolic pathways in biological cells and new synthetic pathways for metabolites and metabolite-like molecules, can provide blueprints and starting points for biocatalytic platform technologies. The interaction of scientific disciplines of classical organic chemistry and biocatalysis in total synthesis, microbial whole-cell and cell-free approaches in design and integration, or reaction/process engineering with protein engineering has allowed a multidisciplinary research community to be established to drive forward the synthesis of metabolite and metabolite-like molecules for their many potential future applications.
